# AI for image quality and patient safety in CT and MRI

**DOI:** 10.1186/s41747-025-00562-5

**Published:** 2025-02-23

**Authors:** Luca Melazzini, Chandra Bortolotto, Leonardo Brizzi, Marina Achilli, Nicoletta Basla, Alessandro D’Onorio De Meo, Alessia Gerbasi, Olivia Maria Bottinelli, Riccardo Bellazzi, Lorenzo Preda

**Affiliations:** 1 Department of Clinical, Surgical, Diagnostic and Pediatric Sciences, University of Pavia, Pavia, Italy; 2 Department of Radiology, IRCCS Policlinico San Matteo, Pavia, Italy; 3 Department of Electrical, Computer and Biomedical Engineering, University of Pavia, Pavia, Italy

**Keywords:** Artificial intelligence, Image processing (computer-assisted), Patient care, Patient safety, Radiation dosage

## Abstract

**Abstract:**

Substantial endeavors have been recently dedicated to developing artificial intelligence (AI) solutions, especially deep learning-based, tailored to enhance radiological procedures, in particular algorithms designed to minimize radiation exposure and enhance image clarity. Thus, not only better diagnostic accuracy but also reduced potential harm to patients was pursued, thereby exemplifying the intersection of technological innovation and the highest standards of patient care. We provide herein an overview of recent AI developments in computed tomography and magnetic resonance imaging. Major AI results in CT regard: optimization of patient positioning, scan range selection (avoiding “overscanning”), and choice of technical parameters; reduction of the amount of injected contrast agent and injection flow rate (also avoiding extravasation); faster and better image reconstruction reducing noise level and artifacts. Major AI results in MRI regard: reconstruction of undersampled images; artifact removal, including those derived from unintentional patient’s (or fetal) movement or from heart motion; up to 80–90% reduction of GBCA dose. Challenges include limited generalizability, lack of external validation, insufficient explainability of models, and opacity of decision-making. Developing explainable AI algorithms that provide transparent and interpretable outputs is essential to enable seamless AI integration into CT and MRI practice.

**Relevance statement:**

This review highlights how AI-driven advancements in CT and MRI improve image quality and enhance patient safety by leveraging AI solutions for dose reduction, contrast optimization, noise reduction, and efficient image reconstruction, paving the way for safer, faster, and more accurate diagnostic imaging practices.

**Key Points:**

- Advancements in AI are revolutionizing the way radiological images are acquired, reconstructed, and interpreted.
- AI algorithms can assist in optimizing radiation doses, reducing scan times, and enhancing image quality.
- AI techniques are paving the way for a future of more efficient, accurate, and safe medical imaging examinations.

**Graphical Abstract:**

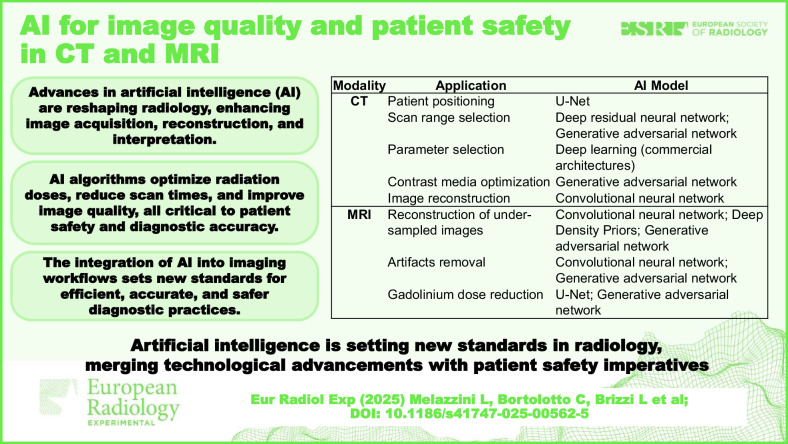

## Introduction

Advancements in artificial intelligence (AI) have significantly transformed clinical radiology by enhancing image quality, reducing scan times, and improving diagnostic accuracy [1]. By optimizing workflows—increasing acquisition efficiency and decreasing radiation exposure—AI not only ensures superior imaging but also enhances patient safety, making it an indispensable tool in modern radiology [2]. As policies like the European Union’s AI Act guide the development of these tools, AI applications in radiology are being designed with a strengthened focus on regulatory compliance and data privacy, aligning innovation with standards for safe, ethical clinical use [3]. This regulatory focus not only promotes trust and safety but also supports the efficient implementation of AI technologies. By improving workflows and reducing inefficiencies, AI adoption has the potential to lower operational costs while delivering high-quality care, making it a cost-effective solution for healthcare systems [4].

In computed tomography (CT) and magnetic resonance imaging (MRI), AI is revolutionizing imaging practices. In CT, AI refines patient positioning [5], optimizes scan parameters [6, 7], and reduces unnecessary use of iodined contrast media (ICM) [8, 9], while advanced algorithms surpass traditional reconstruction methods in denoising and artifacts reduction [10–14]. In MRI, AI maintains image quality even with faster scans, addressing limitations of conventional methods that often compromise image integrity for shorter scan times [15–22], and contributes to lowering the dose of gadolinium-based contrast agents (GBCAs) [23–26]. In this scenario, expanding the potential of AI, generative AI introduces a creative dimension to radiology, enabling the development of high-quality images that complement existing techniques in CT and MRI and contribute to improving imaging workflows [27]. Table 1 summarizes the main AI applications in CT and MRI for image reconstruction and patient safety discussed in the main text.

**Table 1:** Main applications of AI for image quality and patients’ safety in CT and MRI

Application	Modality	AI model
Patient positioning: automated table centering	CT	U-Net [5]
Scan range selection from scout images	CT	Deep residual neural network [6]; GAN [7]
Parameter selection	CT	Deep Learning^a^ [40–42]
Contrast media optimization	CT	GAN [8, 9]
Image reconstruction	CT	CNN [10–14]
Reconstruction of under-sampled images	MRI	CNN [15–17]; Deep Density Priors [18]; GAN [21]
Artifacts removal	MRI	CNN [19, 20]; GAN [22]
Gadolinium dose reduction	MRI	U-Net [23–25]; GAN [26]

*CNN* Convolutional neural network, *GAN* Generative adversarial network

^a^ Model architecture of commercial solutions not specified by the vendors

This narrative review aims to describe applications of AI in CT and MRI that help optimize imaging protocols and improve image quality while minimizing risks associated with radiation and contrast agents. Translating these advancements into clinical practice may foster greater healthcare efficiency while also reducing costs associated with inefficiencies and patient safety risks [2, 28].

## Computed tomography

Computed tomography has long been a cornerstone of diagnostic imaging and continues to be a primary modality in medical imaging [29]. AI offers promising advancements in enhancing its diagnostic image quality while adhering to the “as low as reasonably/diagnostically achievable”—ALARA principle for radiation doses [30]. AI technologies have been developed to optimize each stage of the CT process, from patient preparation to image reconstruction [31]. Recent AI innovations aimed at optimizing ICM use, coupled with advances in deep learning (DL)-based image reconstruction, enable the maintenance of high-quality CT imaging while simultaneously reducing the radiation dose and optimizing ICM administration [30–32].

### Exam preparation

#### Patient positioning

Within the CT system, the x-ray tube-detector pair rotates around a fixed center, namely the “isocenter”. Correct patient positioning is defined by the height of the table at which the isocenter of the patient coincides with the isocenter of the scanner. From the 2000s onwards, an “automatic exposure control” has been incorporated into CT systems, according to the principle that x-ray attenuation and quantum image noise are determined by the size of the object and its tissue density [33]. Automatic exposure control is a tool that varies the number of x-ray photons based on the thickness of the various regions of the body, based on the information provided by the localizer radiograph. If the patient’s positioning is not correct, too high or too low to the isocenter, the system perceives the patient as too thin or too thick and applies an incorrect radiation dose [31].

To address this, AI-based strategies for optimizing patient positioning have been implemented [34]. Some CT manufacturers have integrated a three-dimensional infrared camera placed on the ceiling above the CT table, which detects reference points on the patient’s surface, reproducing their three-dimensional image. A 2019 study evaluated the accuracy of this three-dimensional camera for patient positioning in CT. The camera worked on a statistical shape model to assume the pose and the body proportions of the patient. Automatic positioning by using the camera outperformed manual positioning done by radiographers, with significantly less deviation from ideal table height [35].

In 2023, a method for automatic patient positioning that relied on the deep neural network using only the CT image localizer was proposed. The patient’s body centerline distance from the gantry isocenter was automatically determined to obtain table height and positioning, achieving performance comparable to alternative techniques such as the external three-dimensional camera. Notably, the method had the advantage of being free from errors related to objects blocking the camera visibility [5].

#### Scan range selection

Once the patient is correctly centered in the isocenter, the operator, relying on the localizer radiograph, must select the anatomical portion from which the data will be acquired. This selection in clinical practice is usually done by the operator manually, and the scan length is commonly selected on landmarks extracted from two-dimensional anterior-posterior and/or lateral scout scans [7]. The process is in most cases prone to human error and highly operator-dependent, often resulting in too much or too little anatomical coverage. For instance, it is common to excessively extend the scan length to avoid the possibility of excluding any anatomical structure from the scan. This “overscanning” causes excessive radiation exposure to patients undergoing CT examinations and must be avoided [36]. AI algorithms have been trained to accurately identify human anatomy and choose the scan range optimally centered around the anatomical structures required by exam indication, thus optimizing patients’ radiation dose [37].

Driven by the high number of chest CT scans performed during Covid19 pandemic, a completely automated system for selecting the acquisition range was proposed in 2021 [6]. It was based on a deep residual neural network algorithm that considered exact lung coverage as the ground truth and anterior-posterior and lateral projections as input to provide scan range delimitation with an error of 0.08 ± 1.46 (mean ± standard deviation) and -1.5 ± 4.1 mm in superior and inferior directions, respectively. With the proposed method, the effective dose reduction achieved by automatic scan selection was as high as 21% [6].

In the same year, another method that relied on generative adversarial networks (GAN) was adopted to automatically delineate the scan range based on chest CT topograms, using the scan range annotated by expert radiologists as the ground truth. The algorithm-based scan ranges achieved an absolute difference of 1.8 mm ± 1.9 and 3.3 mm ± 5.6 at the upper and lower boundary, respectively, gaining lower simulated total radiation exposure [7].

### Exam acquisition

#### Parameter selection

During the image acquisition phase in CT, parameters such as tube current and voltage are selected based on patient-specific biological factors, including age, height, and weight. Manual adjustment of these parameters introduces significant risks of interoperator variability and human error, which can lead to suboptimal scan settings [38]. To improve this process and reduce dose exposure, automated systems that account for patient characteristics can be employed alongside technologies such as automatic exposure control [39]. Manufacturers have developed parameter selection systems integrated into their commercial products, where decision algorithms tailor scan parameters based on individual patient characteristics. Once the patient is properly aligned and personal data is retrieved, these systems automatically provide the optimal settings for generating low-dose, high-quality images. These vendor-specific systems are incorporated into the acquisition workflow and are allegedly based on DL technologies [40–42].

#### Contrast media optimization

In recent years, progress has been made in optimizing the dose of ICM through several approaches that can be broadly categorized into those that act directly in the image acquisition phase and those that act in the post-processing phase [43]. The first approach aims at optimizing scan timing, obtaining images at the moment of maximum contrast enhancement in the region at study. The second approach exploits AI techniques to improve the quality of CT scans acquired using a reduced amount of ICM [44].

Two pioneering studies published in 2019 introduced novel algorithms designed to optimize contrast-enhanced CT imaging by tailoring acquisition timing to patient-specific cardiovascular data [45, 46]. An approach was developed to predict the aortic contrast enhancement curve by continuously monitoring the patient’s cardiovascular system after contrast injection, enabling a personalized trigger delay that improved image quality and provided a superior contrast-to-noise ratio compared to fixed delay methods [45]. A bolus tracking software was also implemented to determine the optimal transition delay between reaching the density threshold in the aorta and initiating the scan, using real-time data and empirical enhancement curves from previous patients [46]. This individualized strategy not only enhanced image quality but also reduced the required ICM dose and injection flow rates by optimizing the timing of the scan, minimizing the risk of contrast extravasation [46]. These approaches have the potential to reduce both the total amount of ICM and injection flow rates, thus minimizing the risk of extravasation, while maintaining diagnostic accuracy.

Further opportunities for optimizing contrast media usage have emerged due to advancements in DL-based image post-processing techniques. A GAN was trained and validated to selectively enhance image quality and diagnostic accuracy in CT images acquired with lower doses of ICM. Notably, dual-energy CT was utilized to obtain images with virtually reduced ICM dose levels. The GAN algorithm successfully enhanced ICM-induced image contrast in images with a virtually 50% reduced ICM dose, achieving consistency deemed appropriate for clinical application [8].

Similarly, GANs can be used to enhance diagnostic accuracy using conventional imaging techniques by improving image quality and visibility of key features [47]. A GAN was employed to generate virtual monoenergetic images at 40 keV from conventional contrast-enhanced single-energy CT scans of the upper abdomen. The model achieved a peak signal-to-noise ratio of 45.2, indicating extremely high image quality, significantly improving image quality and lesion visibility as compared to single-energy CT. The algorithm was also tested on patients with hepatocellular carcinoma lesions in the liver, demonstrating that GAN-generated virtual monoenergetic images could enhance diagnostic precision for liver pathologies while utilizing conventional contrast-enhanced CT data [9].

### Image reconstruction

Image quality is closely related to the mathematical transformation of raw data into two- and three-dimensional images. Research has focused heavily on the development of postprocessing algorithms capable of improving the quality of the image obtained while delivering the lowest possible radiation dose [30]. The two most used reconstruction techniques since the introduction of CT are filtered back projection (FBP) and iterative reconstruction (IR), with the latter gradually replacing FBP [48].

FBP is computationally simple but amplifies noise and lacks advanced artifact correction, leading to degraded image quality and diagnostic accuracy in low-dose CT scans. IR addresses these limitations by iteratively refining images using mathematical models, which enhances spatial resolution and suppresses noise; however, IR can increase reconstruction times and may produce images with an unnatural “waxy” appearance when high reconstruction strength levels are used with low-dose acquisitions [48]. Hybrid iterative reconstruction (HIR) is a conventional IR technique that combines features of both FBP and IR to enhance image quality and reduce noise; this technique is less computationally demanding but also less capable of noise and artifact reduction than IR [49].

DL-based CT image reconstruction has emerged as a transformative approach in medical imaging, aiming to enhance image quality while minimizing radiation exposure. By utilizing advanced neural networks, particularly convolutional neural networks (CNNs), these methods learn complex mappings from raw projection data or preliminary reconstructions to high-fidelity images. This approach effectively reduces noise and artifacts commonly associated with low-dose or sparse-view CT scans. Compared to traditional IR techniques, DL models can achieve superior image quality with faster computational times [49].

In 2019, TrueFidelity (GE Healthcare) became the first DL reconstruction technique approved by the FDA. During its training phase, the algorithm used lower-dose sinograms—the raw projection data from the CT scan—obtained from both phantom studies and patient scans as input data. A direct DL reconstruction model employing a CNN generated estimations of the reconstructed images from these sinograms. These estimations were then compared to high-dose images reconstructed using FBP, which served as the ground truth. Because TrueFidelity was trained using FBP images, the output retains FBP-like characteristics, including noise texture, image sharpness, and artifact properties [10].

In the same year, the Advanced Intelligent Clear-IQ Engine (AiCE) by Canon Medical Systems received FDA approval as the second DL reconstruction algorithm. AiCE is based on a CNN trained with patient data where lower-dose HIR images are used as input, and routine-dose full IR images serve as the ground truth. AiCE employs an indirect, image-based DL reconstruction approach that begins with sinogram filtering, incorporating scanner-specific information such as gantry size and detector materials. An initial image is reconstructed using HIR and then fed into the CNN, which outputs an enhanced image. While the reconstruction time per scan for AiCE is slightly longer than that of HIR (44 s *versus* 27 s for brain scans), it remains substantially shorter by a factor of three to five times than the reconstruction times required for full IR [11].

Furthermore, other vendors have developed DL reconstruction algorithms. In 2022, Philips Healthcare released Precise Image, a direct DL reconstruction algorithm approved by the FDA, which employs a CNN trained on lower-dose simulated sinograms with added noise and matches them to routine-dose FBP images serving as ground truth [12]. Additionally, to address the limitations of vendor-specific solutions, vendor-neutral DL–based denoising algorithms such as ClariCT.AI (ClariPi) [13] and PixelShine (AlgoMedica) [14] have been introduced. Specifically, ClariCT.AI showed superior noise reduction, enhanced spatial resolution, and improved overall image quality compared to HIR in lower-dose chest CT scans [50]. Similarly, PixelShine was found to improve noise levels, reduce streak artifacts, and enhance overall image quality when applied to lower-dose chest CT images reconstructed with FBP, HIR, and model-based IR techniques [51].

## Magnetic resonance imaging

Most AI techniques in radiology have focused on the use of AI solutions for the detection of anatomical structures or lesions and subsequent image segmentation, with neuroradiology being the most common subspecialty for their application [52]. These tools are well-integrated into medical practice and currently support radiological reporting and clinical decision-making [52, 53]. The same cannot be said for AI-based algorithms for image acquisition, MRI reconstruction, and image quality. In this setting, CNNs play a major role among varying DL-technique [53], and applications are currently being proposed with the purpose of reducing the length of MRI protocols, lowering GBCAs dose, performing image harmonization, improving image quality and removing artifacts [54]. In this framework, DL has the opportunity to make an impact on such challenges, with fewer ethical issues and medico-legal risks compared to post-processing tools that guide treatment decision-making [55].

### Reconstruction of undersampled images

In MRI, raw k-space data must be transformed into the image domain for clinical interpretation, a process that is often time-consuming. Traditional methods to reduce acquisition time, such as varying k-space trajectory, parallel imaging with multiple coils, or compressed sensing with sparse representations, can be effective but typically sacrifice image quality [56]. Emerging AI applications now complement these methods, potentially preserving or even enhancing image quality in undersampled datasets [15].

**Fig. 1:**
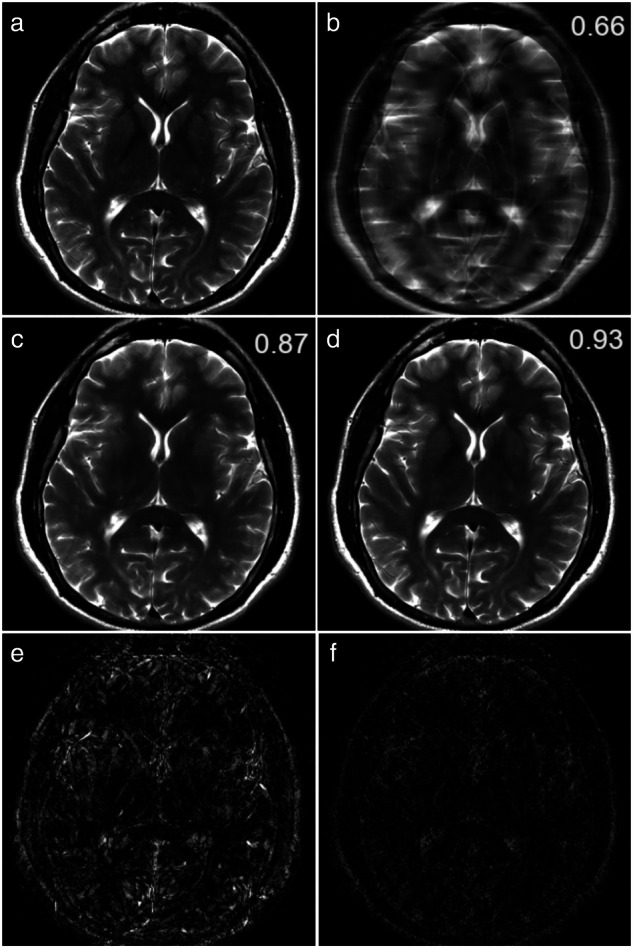
Results of CNN-based algorithms for image enhancement that work on either raw data or on already-reconstructed images. **a** Brain T2-weighted reference image, axial scan at the basal ganglia level, fully sampled. **b** Four-times accelerated image, zero-filled. **c** Output from the postprocessing algorithm. **d** Output from the raw-data algorithm. **e** Absolute errors for panel (**c**). **f** Absolute errors for panel (**d**). The structural similarity index (range 0–1) is displayed in white at the top right of every image. Reproduced with permission [15]

Most AI-based reconstruction and denoising algorithms fall into two groups: those that work directly on raw (often multicoil) data and postprocessing methods that use reconstructed images as input. The former leverages a richer dataset, including phase and coil sensitivity data, while the latter, using the Digital Imaging and Communications in Medicine−DICOM standard protocol, benefits from simplicity and a larger pool of training data [15]. Figure 1 illustrates that reconstruction methods based on raw data achieve higher structural similarity index measure values—ranging from 0 (no similarity) to 1 (perfect similarity)—compared to postprocessing models, showing superior detail retention and less blurring in four-times accelerated T2-weighted images [15].

DL-based reconstruction of undersampled images that work on raw data generally requires paired datasets in the training phase, where prior information on the structure of fully-sampled images is needed to compensate for missing k-space data in the corresponding under-sampled image. A new approach to this subject was introduced [18], using unsupervised DL to learn the probability distribution of fully-sampled MRI images, and this prior information was utilized in reconstruction, without requiring paired datasets for training. This method was evaluated on a publicly available dataset of brain T1-weighted images, scans from healthy controls, and brains with a high burden of white matter lesions. The unsupervised DL algorithm showed high-quality reconstructions and outperformed most of the available methods on the same dataset. According to the authors, their method has the advantage of being less dependent on acquisition specifications and coil settings, and can theoretically be applied to reconstruct any sampling scheme without the need for algorithm retraining [18].

Dynamic imaging poses a major challenge for the application of DL-based reconstruction methods on sparsely sampled data. In this setting, cardiac dynamic sequences take the lion’s share. In cine MRI the pattern of pixels out of the heart remains consistent across time. Moreover, the signal of pixels in the heart for any acquisition is strictly correlated to the previous and succeeding signal in that very pixel location. A network that efficiently leverages such spatiotemporal redundancy would boost its performance and achieve more aggressive undersampling compared to DL architectures that work on static images. Using this technique, missing k-space data for each frame can be filled using the samples from adjacent frames. This principle (named the “data-sharing” approach) combined with convolution has been exploited to develop recurrent CNNs that are able to reconstruct cardiac MR images that are up to eleven times undersampled [16, 17].

GANs offer significant advancements in MRI reconstruction, particularly for compressed sensing and de-aliasing from incomplete k-space data [57]. RefineGAN, an advancement over earlier GAN models, was proposed to incorporate cyclic consistency, ensuring that transformed images could revert to their original state, and residual learning, allowing the network to focus on targeted corrections to specific image features rather than recreating the entire image. This model achieved high image quality even at extremely low sampling rates (10%) [21]. By efficiently reconstructing high-quality images from limited data, these GAN-based methods can significantly reduce acquisition times and enhance diagnostic accuracy, making GANs a highly promising tool for rapid, clinically applicable MRI reconstructions [57].

### Artifacts removal

Motion artifacts compromise image interpretability and can lead to radiological misdiagnosis [15, 58, 59]. Motion correction techniques are applied either prospectively, during acquisition, or retrospectively [58, 59]. Prospective correction maintains constant spatial alignment between scanning coordinates and the target region by real-time tracking of position and orientation, enabling the imaging volume to adaptively “follow” the region of interest [59]. However, this approach may reduce scanning efficiency due to repeated acquisitions of motion-corrupted data. Retrospective correction, by contrast, relies on computational processing during reconstruction, thus preserving scan efficiency [58].

Notably, data-driven retrospective methods do not require sequence modifications or external tracking devices with consequent little impact on the clinical workflow, and AI showed promising results for implementation in retrospective motion correction methods for its ability to increase computation efficiency [60]. An artifact removal CNN that integrated a model-based motion estimation was introduced [19]. The CNN motion removal performance was tested on both simulated motion-corrupted images and brain scans affected by intentional head shaking. The network was able to minimize motion artifacts across every image from previously unknown datasets, showing full potential for efficient post-acquisition motion correction in real-world datasets [19].

Meanwhile, among applications of DL-reconstruction techniques for dynamic cardiac imaging, a DL algorithm that automatically identified and corrected motion-related artifacts in cardiac MRI acquisitions during reconstruction from k-space data was presented [20]. The method specifically spotted corrupted k-space lines due to mistakes in electrocardiogram-triggering, dysrhythmia, or patients’ motion. Once the corrupted lines were correctly identified and removed, a recurrent CNN—such as the one developed in [17]—was applied to accomplish the image reconstruction task. The method was evaluated on good-quality 300 cine steady-state free precession acquisitions and synthetic motion-corrupted images from the United Kingdom biobank database. The proposed algorithm impressively reduced the impact of k-space corruption and outperformed other state-of-the-art motion removal methods [20].

A recent study proposed a GAN-based model for motion artifact correction specifically in fetal MRI, where motion artifacts are particularly problematic due to fetal and maternal movement [22]. The model’s generator network follows an autoencoder structure that compresses and then reconstructs images, effectively isolating and removing artifacts. Additionally, it incorporates squeeze-excitation blocks, which enhance important features in the image by dynamically adjusting the emphasis on relevant details, helping to reduce noise without losing key anatomical information. Trained on both synthetic and real motion-corrupted images, the model outperformed standard methods, achieving a high structural similarity index measure (93.7%) and a peak signal-to-noise ratio of 33.5 dB, further supporting GANs’ effectiveness for clinical-quality MRI reconstruction [22].

### GBCA dose reduction

The use of GBCAs has been generally recognized as safe, except for rare mild adverse reactions, such as hypersensitivity, nausea, and chest pain [61]. However, studies have demonstrated deposition of gadolinium in the dentate nucleus and globus pallidus in the brain, especially in patients who undergo repeated contrast-enhanced MRI acquisitions [62]. Also, the use of GBCAs in patients with deteriorated renal function moderately increases the risk of developing nephrogenic systemic fibrosis, a potentially life-threatening condition [63]. Safety concerns in the use of GBCAs in the medical community have placed emphasis on the development of AI-based methods that could potentially reduce contrast dose while preserving image quality and the information provided by a full-dose contrast scan [64].

**Fig. 2:**
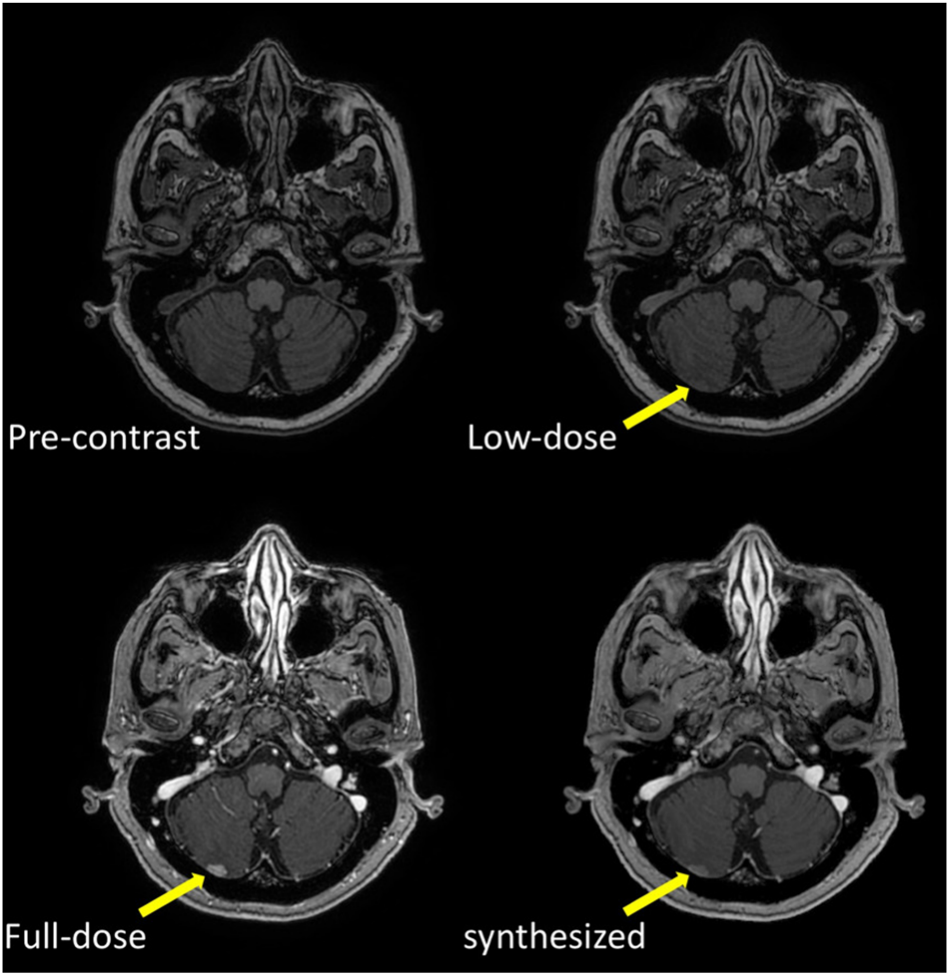
Illustrations of a precontrast, full-dose (100% contrast), low-dose (10% contrast), and synthesized full-dose (10% contrast) T1-weighted brain scan. The figures show the case of a patient with intracranial metastatic disease. Reproduced with permission [23]

In 2018, a DL model was developed to approximate full-dose brain images from pre-contrast and low-dose images [23]. The workflow included acquiring three scans—precontrast T1-weighted, low-dose (10%), and full-dose (100%) postcontrast T1-weighted), preprocessing (coregistration and normalization), and training the DL model. In the U-net-based network, the full-dose scan served as ground truth, while pre-contrast and low-dose scans were inputs, yielding an output approximating the full-dose MRI. The technique assumed low-dose MRI to be a scaled, noisier version of the full-dose scan. Expert neuroradiologists rated the synthesized images as comparable to full-dose images in quality and diagnostic utility, with non-inferiority testing confirming image quality, artifact suppression, and contrast enhancement at a GBCA dose ten times lower than the one typically used (Fig. 2) [23]. An upgraded version of the model was released in 2021, incorporating data from three different sites and scanners [24]. This updated model achieved high similarity metrics with the full-dose scans, with a structural similarity index measure of 92% and a peak signal-to-noise ratio of 35.1 dB, compared to 85% and 28.1 dB in the initial study [23, 24].

A similar approach was implemented for MRI of the heart and great vessels in congenital heart disease imaging, proving particularly beneficial for young patients who require repeated acquisitions throughout their lifetime. In a 2021 study [25] low-dose images were acquired using 20% of the full contrast dose, followed immediately by the remaining 80% to obtain a full-dose scan. Virtually-enhanced low-dose images using a variant of the U-net architecture were comparable to full-dose images in terms of diagnostic confidence, edge sharpness, perceptual contrast, agreement of manual measurement of vessel diameters, signal-to-noise ratio and contrast-to-noise ratio [25].

Furthermore, a recent study compared the efficacy of GANs and diffusion probabilistic models in enhancing breast MRI images acquired with reduced GBCA doses [26]. Using contrast subtraction images at 5%, 10%, and 25% of the typical contrast dose, the study found that GANs provided superior reconstruction quality at the lowest dose (5%), while diffusion probabilistic models performed better at higher doses (25%). Both models showed clinical promise in reducing GBCA dose requirements while maintaining diagnostic image quality, highlighting the potential of GANs in optimizing contrast enhancement protocols across varying dose levels [26].

## Challenges, future directions, and conclusions

Current challenges in AI for CT and MRI include limited generalizability, as many algorithms are tailored to specific scanner models and acquisition parameters [65–67], as well as the lack of sufficient and externally validated datasets, which raises significant concerns about their reliability in clinical practice [68–70]. Expanding training datasets and conducting real-world validation on independent data are critical steps to ensuring robust and reproducible results [71]. Another major barrier is the insufficient explainability of AI models and the opacity of their decision-making processes, which undermine trust and hinder their adoption in clinical workflows [68]. Developing explainable AI algorithms that provide transparent and interpretable outputs is thereby essential to overcoming these challenges and enabling seamless integration into routine practice [72].

To summarize, AI has emerged as a transformative force in CT and MRI, enhancing image quality, optimizing workflows, and improving patient safety [1, 73, 74]. With the current state-of-the-art equipment and AI techniques, it will be possible to obtain super-specific examinations in the future, interpret them, and store them free of unnecessary data, holding immense potential for improving healthcare outcomes [75]. However, realizing this potential requires not only technical progress but also dedicated funding to integrate AI-based technologies into clinical practice [74]. At the same time, recent policies like the European Union’s AI Act are ensuring that these tools meet rigorous safety and ethical standards, creating a solid foundation for their adoption. These advancements are paving the way toward a future where precision, efficiency, and patient-centered care define the next generation of medical imaging.

## Data Availability

The two figures included in this manuscript are extracted from previously published studies. Permission for reproduction was obtained through the CCC Copyright Clearance Center.

## Abbreviations

AI: Artificial intelligence
AiCE: Advanced intelligent Clear-IQ Engine
CNN: Convolutional neural network
DL: Deep learning
FBP: Filtered back projection
GAN: Generative adversarial network
GBCA: Gadolinium-based contrast agent
HIR: Hybrid iterative reconstruction
ICM: Iodined contrast media
IR: Iterative reconstruction
